# Predicting Overall Survival Time in Glioblastoma Patients Using Gradient Boosting Machines Algorithm and Recursive Feature Elimination Technique

**DOI:** 10.3390/cancers13194976

**Published:** 2021-10-04

**Authors:** Golestan Karami, Marco Giuseppe Orlando, Andrea Delli Pizzi, Massimo Caulo, Cosimo Del Gratta

**Affiliations:** 1 Department of Neuroscience, Imaging and Clinical Sciences, Gabriele D’Annunzio University, 66100 Chieti, Italy; marco.g.orlando@asl.taranto.it (M.G.O.); andrea.dellipizzi@unich.it (A.D.P.); massimo.caulo@unich.it (M.C.); cosimo.delgratta@unich.it (C.D.G.); 2 Institute for Advanced Biomedical Technologies, Gabriele D’Annunzio University, 66100 Chieti, Italy

**Keywords:** GBM, gradient boosting, random forest, recursive feature elimination, survival time

## Abstract

**Simple Summary:**

Despite the highly aggressive nature of glioblastoma multiforme (GBM), survival time is in practice highly variable, and some of the patients remain stable for several years after treatment. The aim of this study was to develop a machine learning method that could precisely predict survival time of GBM patients. To do so, we integrated multi-modal MRI with non-supervised and supervised machines. We first identified compartments of the tumor then extracted their features. Then relevant useful features were selected by Random Forest-Recursive Feature Elimination (RF-RFE) to feed into Gradient Boosting Machine Algorithm with the aim of classifying GBM patients. By selecting the most relevant features, multi-modality MRI with tumor segmentation provided valuable independent and complete features to feed a machine learning model. Additionally, advanced machine-learning methods such as RF-RFE and GBoost are powerful tools for data mining. Hand-crafted feature-based methods have shown promising results, but there is no systematic way to determine survival-related hand-crafted features and existing methods mostly rely on experience.

**Abstract:**

Despite advances in tumor treatment, the inconsistent response is a major challenge among glioblastoma multiform (GBM) that lead to different survival time. Our aim was to integrate multimodal MRI with non-supervised and supervised machine learning methods to predict GBM patients’ survival time. To this end, we identified different compartments of the tumor and extracted their features. Next, we applied Random Forest-Recursive Feature Elimination (RF-RFE) to identify the most relevant features to feed into a GBoost machine. This study included 29 GBM patients with known survival time. RF-RFE GBoost model was evaluated to assess the survival prediction performance using optimal features. Furthermore, overall survival (OS) was analyzed using univariate and multivariate Cox regression analyses, to evaluate the effect of ROIs and their features on survival. The results showed that a RF-RFE Gboost machine was able to predict survival time with 75% accuracy. The results also revealed that the rCBV in the low perfusion area was significantly different between groups and had the greatest effect size in terms of the rate of change of the response variable (survival time). In conclusion, not only integration of multi-modality MRI but also feature selection method can enhance the classifier performance.

## 1. Introduction

Glioblastoma multiforme (GBM) as high-grade gliomas (HGGs), is the most common and aggressive brain malignancy in adults, consisting of 16% of all primary central nervous system neoplasms [1], with a median survival of 15 months [2]. Despite the highly aggressive nature of GBM, some of them remain stable for several years after treatment, and their prognosis and survival times are practically different [3].

Studies indicated that traditional WHO grading could not capture the biological characteristics of gliomas and lacks power in prognosticating the clinical course of gliomas. The 2016 CNS WHO presented major restructuring of the diffuse gliomas classification, and for the first time, used molecular parameters in additions to histology [4]. The WHO classification of CNS tumors was defined by both histology and molecular features, including glioblastoma, IDH-wild type, and glioblastoma, IDH-mutant. The molecular subtypes depend on the presence or absence of mutations in the isocitrate dehydrogenase (IDH) gene. To better predict survival time for gliomas patients, emphasis is put on the identification of molecular phenotypes [5,6]. Gliomas patients with IDH-mutated survive longer than those with IDH-wild type [7]. The 2021 fifth edition WHO classification introduces major changes that advance the role of molecular diagnostics in CNS tumor classification. GBMs are classified as adult type diffuse gliomas patients [8].

Healthcare has focused on improving survival in the treatment of brain tumor patients [9,10]. The effectiveness of the treatment procedure depends on the extent of resection and sensitivity of the tumor to chemo-radiation therapy, molecular subtype of tumors, and their grading [7,11]. Despite the advances in treatment, the inconsistent response is a major challenge in treatment, which could be related to the extensive heterogeneity [12], and to molecular subtype [13]. Now the only way to identify a molecular diagnostic of gliomas is through surgical biopsy or complete resection of the tumor. Since, surgical biopsy often provides limited information due to tumor spatial heterogeneity and does not allow real-time monitoring of the tumor, and GBMs can infiltrate the surrounding brain tissue rapidly, early non-invasive diagnosis in order to design individual treatment planning is critical. Moreover, a variety of distinct cells exist in the tumor which display diverse treatment responses which can be the key of treatment failure [12].

Magnetic resonance imaging (MRI) is a rich source of patients’ information, and being used as a part of the procedure for diagnosis and follow-up of brain tumor patients [14]. Moreover, MRI techniques including dynamic susceptibility contrast (DSC) and diffusion tensor imaging (DTI) provide a valuable source of data to evaluate more comprehensively the biology of the tumors. Diffusion and also perfusion differ within the tumor, and differences of patients’ survival might be caused due to differences in this heterogeneity. DSC-MRI allows investigating tumor angiogenesis and perfusion. Due to high angiogenesis of GBMs, they show high perfusion and intra-tumor heterogenous vascular pattern with a necrotic area. When the demand and supply of nutrients are mismatched the sufficiently perfused habitats may lead to progression and proliferation, whereas the insufficiently perfused habitats may induce clones resistant to therapy via hypoxia. Hence, this heterogeneity could cause poor treatment and inconsistent response [15,16]. DTI-MRI provides information about the motion of water protons at the cellular level reflecting structural data on cellular density and extracellular space [17]. The mean diffusivity (MD) is a MRI biomarker that correlates with cellular packing [18] and glioma grade [19,20]. Fractional anisotropy (FA) values describe the degree of anisotropy in the given voxel. Anisotropy reflects the increased cellularity and restriction of water diffusion in high-grade gliomas [21,22,23].

The parameters derived from DTI and DSC could represent complementary information of GBM patients, but due to the large number of features and the comparatively low number of patients, a preliminary feature selection method to better classification is required. Hence, not only the useful selected features may improve data’s compatibility with a machine learning model class but also it will shorten training time.

Hand-crafted features for machine learning techniques have been explored to predict patient outcomes. Vergun et al. [24] used multimodal images including resting-state fMRI, task fMRI, and DTI in combination with clinical and demographic variables fed into a support vector machine (SVM) to predict outcomes. One of the predicted variables was mortality within 18 months after surgery. Their model was able to classify patient mortality with 80.7% accuracy. In another study using multimodal MRI (post-contrast T1 and DTI), Nie et al. [25] proposed a multi-channel architecture of 3D convolution neural network (CNN) to extract high-level features. Then these features along with demographic tumor features were fed into SVM to finally predict overall survival time. They obtained 90.66% accuracy in predicting survival time. Chaddad et al. [26] proposed a novel class of multimodal image features based on the joint intensity matrix (JIM) to model fine-grained texture signatures in the radiomic analysis of low-grade glioma (LGG) tumors to predict gene status and overall survival time of patients. Their results showed that JIM features can predict the mutant or wild-type status of relevant genes for LGG. Classification combining all features including volume, JIM, and gray-level co-occurrence matrix (GLCM) resulted in an AUC value of 86.79% in predicting short and long LGG patient survival outcomes. Moreover, they showed that JIM features were generally the most informative predictors and provided information that is complementary to conventional GLCMs.

Recently Recursive Feature Elimination (RFE) algorithm appeared as one of the most popular feature selection techniques which provides good performance in many applications [27,28,29]. RFE uses a classifier to rank the features and recursively removes the weakest features [30,31]. The process of removing the weakest features continues until the number of required features is reached. Random Forests (RF) algorithm as a classifier adds randomness in selecting a subset of predictors and the output of the random forest is the class selected by most trees. Due to this randomness in the feature selection, the RF classifier works more robustly compared to other classifiers such as SVM, discriminative analysis, and neural networks. The RF-RFE estimates which features are more effective to discriminate the classes and eliminates features that are not useful in predicting the class. The performance of RFE depends on the algorithm used to choose features and the number of the features to select. These hyper parameters can be optimized.

Gradient boosting (GBoost) is a powerful ensemble machine learning method [32,33]. The idea of boosting is to use a forward stage-wise strategy to build an additive model. Boosting as an ensemble technique corrects the performance of prior models, and uses the residual errors of the previous model as the weight values. To optimize the performance of the classifiers, learning rate and loss function are changed like Neural Networks. Due to features such as being user friendly and impressive predictive accuracy, GBoost has shown to perform exceptionally well in different fields. Ogunleye et al. modeled extreme gradient boosting (XGBoost) method for Chronic Kidney Disease (CKD) diagnosis and achieved high accuracy and performance [34]. Zhong et al. applied XGBoost method and showed that incorporating multivariate features improved the performance of the method in predicting protein [34].

Despite the comprehensive advanced imaging, there is still uncertainty about GBM survival time. In this study, we exploited the potential of multi-modal MRI and GBoost machine to address the issue of predicting survival time of GBM patients. We used multi-modality data (post-contrast T1w, T2-w, DTI, and DSC maps) and determined different compartments of the tumor. We hypothesized that survival differences of patients might be caused due to differences in GBM tumor heterogeneity, and perfusion and diffusion parameters derived could represent complementary information of GBM patients. Intensity and texture features of different tumor compartments play an important role in the creation of biomarkers for gliomas and might be a specific marker of patient outcome. Moreover, in order to select the main relevant features, we used RF-RFE method. We expected these features to facilitate and complement the machine learning framework to predict GBM patient survival time.

## 2. Materials and Methods

### 2.1. Patients Cohort

Neuroimaging and clinical data of 29 Glioblastoma multiforme (GBM) patients with known survival time were included in this study. These patients were selected from a database of individuals who received DSC and DTI as part of presurgical planning for gliomas patients at ITAB, Università degli Studi “G. d’Annunzio” Chieti-Pescara between May 2011 and August 2016. This study was approved by the local ethics committee. Patients signed informed consent on use of their data. All patients underwent surgery, and tumors were histologically proven. Exclusion criteria included low grade patients, history of previous cranial surgery, and inability to undergo MRI scanning. GBM patients with available DSC data, DTI, and survival data were analyzed.

Table 1 lists the clinical information of the patients. The collected clinical data included age, gender, date of surgery, histology and WHO tumor grade, and date of death or date of last follow-up, in some cases there was molecular information as well. All patients underwent complete tumor resection surgery. The survival times were defined as the lapse between the date of pre-surgery MRI and the date of death or last follow-up.

The prognosis for GBM patients is poor, and median survival is 15 months [1] even with an advanced treatment. Therefore, we chose less than 15 month as short-term survival time, and more than 15 month as long-term survival time to classify GBM patients.

### 2.2. MR Imaging Protocols

All MR images were obtained preoperatively with a 3-Tesla MR imaging scanner (Achieva, Philips Medical Systems, Amsterdam, the Netherlands) with 8 channel head coil. The preoperative MRI sequences included post-contrast T1-weighted 3D volumetric sequences (TR ms/TE ms, 7.7/3.7; slice thickness 2 mm; voxel size, 0.97 × 0.97 × 2 mm^3^; acquisition matrix, 256 × 256 × 70), T2-weighted turbo spin echo sequence (TR ms/TE ms, 3000/80 ms; slice thickness 4 mm; acquisition matrix, 560 × 560 × 27), FLAIR imaging without contrast (TR ms/TE ms/IT ms, 11,000/125 ms; slice thickness 5 mm, acquisition matrix, 560 × 560 × 27), DTI scans were acquired using a pulsed gradient spin-echo sequence with a single-shot echo-planar acquisition(TR ms/TE ms, 4.5/0.72; acquisition matrix: 128 × 128 × 60; slice thickness 2 mm with b-value of 1000 s/mm^2^ in 17 uniformly distributed directions), and DSC scans were acquired using a pulsed gradient spin-echo sequence with a single-shot echo-planar acquisition(TR ms/TE ms, 1720/35 ms; flip angle 75 degrees; slice thickness 5 mm; acquisition matrix 128 × 128 × 25; 50 volumes).

### 2.3. Methodology

The proposed approach consisted of four steps: step 1: Pre-processing-DTI and DSC metrics extraction, co-registration and defining the tumor; step 2: tumor segmentation by K-means clustering; step 3: histogram feature extraction and texture feature extraction using GLCM; step 4: feature reduction using RF-RFE; step5: statistical analysis; step6: training a GBoost machine by applying leave-one-out cross validation approach to evaluate the model.

**Figure 1:**
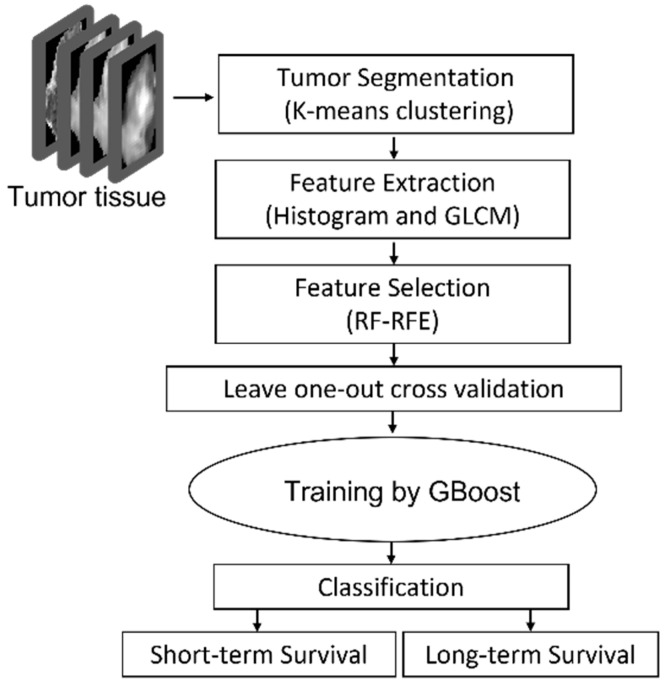
GBMs survival prediction model outline using RF-RFE GBoost machine.

The outline of the proposed approach is illustrated in Figure 1. As shown, the features were extracted from MRIs using histogram and GLCM. After normalization, RF-RFE was applied to derive reduced, discriminated, and uncorrelated set of features. Finally, the reduced features were used as the input of the GBoost machine. The gradient boosting machine is using decision trees and is a powerful ensemble machine learning algorithm. All steps are explained in detail in the following subsections.

#### 2.3.1. Data Pre-Processing

DTI images were processed with the diffusion toolbox in FMRIB Software Library (https://fsl.fmrib.ox.ac.uk (accessed on 1 March 2019)). Eddy current correction, brain extraction, and diffusion tensor fitting were performed, and the FA and MD maps were calculated for each patient.

DSC images were processed by perfusion mismatch analyzer (PMA) software. The arterial input function was automatically defined. A standard population-based arterial input function was defined in PMA software, and maps of cerebral blood volume (CBV) and cerebral blood flow (CBF) were calculated for each patient.

To ensure the same view of physiological and structural images and extract all map features at all tumor slices, images were co-registered to the DTI-B0 images by Linear Image Registration Tool (FLIRT) in FMRIB Software Library (FMRIB, Oxford, UK).

Contouring of the region of interest was performed manually by a neuroradiologist. Post-contrast T1, FLAIR, and T2-weighted images were used to contour the tumor margins manually in 3D-Slicer (https://www.slicer.org (accessed on 1 March 2019)). Care was taken that the tumor mask enclosed entirely the whole solid part of the tumor. To do so, the mask of the tumor was drawn on each slice where the contrast-enhanced on the post-contrast T1-w images and high signal intensity on the T2-w images were visible. The tumor mask was used to extract the post-contrast T1-w, T2-w, MD, FA, rCBV, and CBF maps of the tumor and extract voxel-wise values of them.

#### 2.3.2. Tumor Segmentation by K-Means Clustering

Post-contrast T1, MD, and CBV maps of tumor lesions were segmented by the k-means clustering segmentation method separately. A cluster refers to a collection of data points that have certain similarities, and each data point is allocated to one cluster with the closest similarity.

**Figure 2:**
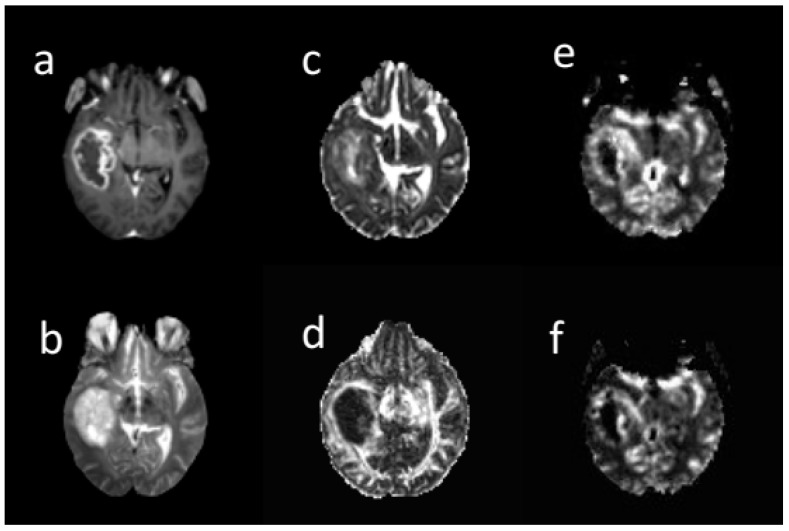
A sample of different brain MRI images: (**a**) post-contrast T1-weighted, (**b**) T2-weighte, (**c**) MD map, (**d**) FA map, (**e**) CBV map, (**f**) CBF map in 66-years-old woman with glioblastoma. All images are co-registered to the B0 DTI images. The tumor region was segmented into contrast enhanced, non-enhanced, necrosis in post-contrast image, low and high diffusion in MD map, and low and high perfusion in rCBV map by k-means. Based on their overlapping, sub-regions were obtained.

Three extracted clusters within post-contrast T1 tumor images included the contrast-enhanced (CE), non-enhanced (non-en), and necrotic. Two clusters including low mean diffusivity (LMD) and high mean diffusivity (HMD) were extracted on the MD tumor map. A low MD cluster was obtained from the lower MD voxels. The low rCBV (LrCBV) cluster was obtained from the lower rCBV voxels (Figure 2). Based on the overlapping different regions, LMD-LrCBV ROI was yielded within the CE region. Moreover, LMD-LrCBV ROI in necrosis region was extracted. A common area between high mean diffusion and low perfusion was defined as HMD-LrCBV within the necrosis ROI.

Iteration of these processes slice by slice was performed on post-contrast T1, the MD, and rCBV tumor maps. Normal-appearing white matter (NAWM) was drawn from the contralateral normal brain tissue individually. This region was far from the tumor location and had no perceivable abnormalities. All these processes were done in MATLAB (R2019b, The MathWorks, Inc., Natick, MA, USA) under the supervision of a neuroradiologist.

#### 2.3.3. Histogram and Texture Feature Extraction and Feature Reduction

For complex feature extraction, we utilized multi-modal MRI to determine different compartments (ROIs) of the tumor and then extract their features. For each individual ROI, three different feature types were extracted.

First, ROI-histogram-based statistics features including mean value, standard deviation, skewness, and kurtosis were calculated. Second, ROI-based texture features of the tumor including contrast, correlation, and homogeneity were calculated. We extracted texture features of the tumor using the gray level co-occurrence matrix (GLCM). The GLCM functions characterize the texture of an image by calculating how often pairs of pixels with specific values and in a specified spatial relationship occur in an image [35]. We analyzed the entire tumor volume and relative volumes of ROIs. Lastly, 480 extracted features were fused to be fed into the machine.

Decreasing the dimension of features is a necessary step before training a machine-learning model to improve the prediction accuracy, the generalization ability, and decrease the computation time as well. In this paper we used RFE as a feature selection approach. There are two configuration options; the choice in classification algorithm to rank the features and recursively remove the lowest features, and the choice in number of features. Hence, we applied Random Forest-based recursive feature elimination (RFE) feature selection for mining 60 best features.

#### 2.3.4. Statistical Analysis

All statistical analyses were performed using SPSS (version 25). After Kolmogorov–Smirnov test, the features were compared by Kruskal Wallis test between ROIs and the statistical significance was evaluated. Features were significantly different between different ROIs. Spearman rank correlation was used to model the relation between the features and survival. Results showed that there is a negative correlation between rCBV and MD mean values with survival (*p* < 0.01). Cox proportional hazards regression analyses were performed to evaluate the features and their effect on survival time.

#### 2.3.5. Gradient Boosting Machine

There are hyper parameters that affect model performance, such as learning rate, size of the data sample used to train each model, and finally depth of the decision tree. We optimized the hyper parameters on the dataset and then trained the model.

We used leave-one-out cross-validation to verify the predictions and avoid overfitting. The advantage of leave-one-out cross-validation is that all observations are used for both training and testing once. LOOCV results were averaged to produce a single estimation to evaluate the testing dataset. Trail LOOCV was repeated 50 times. Finally, metrics, including accuracy, precision, recall, F1-measure, and Matthews correlation coefficient were calculated and reported. We used Scikit-learn, a machine learning package in the python programming language to implement all the codes.

## 3. Results

In this study, we used presurgical neuroimaging and clinical data of 29 GBM patients (19 men, 10 women; mean age, 57.6 years; age range, 23–80 years) with high grade gliomas, 16 short-term and 13 long-term survivals (Table 1). We used post-contrast T1, the MD, and CBV maps to extract regions of interest (ROIs). Histogram-based and texture features were extracted at selected ROIs. After measuring potentially important parameters we fed them to the prediction model. First, we report the statistical analysis results related to the survival time and the features, and then we report the RF-RFE GBoost machine classifier results.

### 3.1. Survival Analysis

The survival times were divided into two groups, short-term (less than 15 month), and long-term (more than 15 month) survival. The short-term overall survival rate was 0.55. The average survival time was 27.1 months, and the median survival time was 13.1 months.

Histogram-based and texture features were compared between group ROIs and groups. Kruskal–Wallis test was used to compare features at the ROI level. Mean value of features were significantly different. Spearman rank correlation was used to model the relation between the features and survival. Post-contrast T1, T2, rCBV revealed a weak negative correlation coefficient with survival time (*p*-value < 0.05).

Univariate and multivariate Cox regression analysis was used to evaluate the features and their effect on survival time. The rCBV in LMD-LCBV in CE region was significant in univariate and multivariate Cox regression analysis (Table 2) and had the greatest effect size in terms of the rate of change of the response variable (survival time).

### 3.2. DTI-DSC Correlation at the Contrast-Enhanced Tumor Region

We found only one significant inverse correlation between derived metrics at the contrast-enhanced region with survival: low rCBV at the CE region (*p*-value < 0.05). The higher rCBV in LMD-LrCBV ROI in CE region was associated with worse survival (HR = 1.433, *p*-value < 0.018). Short-term survival group in the LMD-LrCBV ROI showed higher T2w, rCBV and lower MD values than long-term survival group.

### 3.3. DTI-DSC Correlation at the Necrosis Region

At the necrotic region, we found statistical significance of four derived metrics with survival. Higher rCBV, post-contrast T1w, and T2w values in the necrotic region wereassociated with worse survival (*p*-value < 0.05). Higher rCBV in LMD-LrCBV ROI in the necrotic region was associated with worse survival (HR = 1.028, *p*-value < 0.05). Short-term survival group showed a higher rCBV than long-term survival group (HR = 1.212, *p* < 0.05), and an increased CBF (HR = 0.99, *p* < 0.05).

### 3.4. Texture Features Analysis

Texture features were different according to ROI and survival time. Among these features, contrast on post-contrastT1w images and rCBV in LMD-LrCBV ROI was significantly different between groups (*p*-value < 0.05). Higher post-contrast T1w, FA, and rCBV contrast were associated with worse survival.

### 3.5. Volume Analysis

Table 3 shows the relative volume of ROIs in two groups. There was no significant difference in whole tumor volume between the two groups, whereas there was positive correlation between the necrosis and HMD-LrCBV relative volumes with survival. The results showed that in short-term survival group, the LMD-LrCBV relative volume (3.2 ± 0.73 cm^3^) was significantly larger than LMD-LrCBV relative volume (2.44 ± 0.88 cm^3^) in long-term survival group (*p* < 0.05). Although non-significant, the contrast-enhanced (CE) relative volume was larger in the short-term survival group.

### 3.6. GBoost Survival Classifier Results

**Figure 3:**
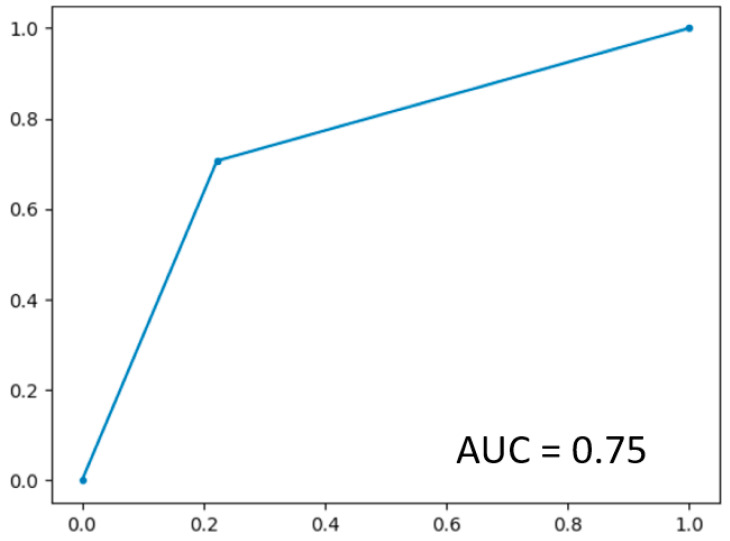
ROC curve on the dataset.

**Figure 4:**
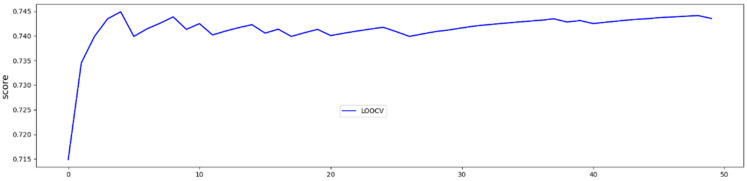
50 trials and averaged test accuracies for each LOOCV trial.

In LOOCV, the GBoost classifier outputs a decision value to predict the survival time class for each single patient, the receiver operating curve (ROC) was delineated for testing datasets generated by the LOOCV (Figure 3). To illustrate the diagnostic performance of the predictive model, the area under the curve (AUC) was calculated (Figure 4, Table 4). A GBoost trained model using best selected features by RF-RFE method was able to classify patient survival with 75% accuracy. Classification of survival time with all features without RF-RFE achieved 58% accuracy (Table 4).

## 4. Discussion

Conventional MRI protocols are regularly used as part of the diagnosis and treatment assessment procedure of gliomas. These images provide a rich source of information for clinical evaluation and statistical predictions. The non-invasive advanced images with conventional MR images may potentially improve the diagnostic value of MR images. Here we integrated conventional and advanced MRI to identify different compartments of diffusion and perfusion of the tumor and then extracted the features. We expected that perfusion and diffusion features derived can represent complementary information of GBM patients, and these features facilitate and complement the machine learning framework. Moreover, we utilized the potential of RF-RFE GBoost machine to predict survival time of GBM patients precisely. Advanced machine learning methods such as RF-RFE and GBoost are powerful tools for data mining

Many studies have assessed the clinical importance of the advanced MRI markers previously. The rCBV as a biomarker of angiogenesis [36] has been positively correlated with tumor vascularity and cellular proliferation [37]. Our finding showed that identifying the tumor components by combined conventional and advanced MRI resulted in an important clinical diagnosis. Our results showed that higher rCBV values in the low perfusion ROI were associated with worse survival (*p*-value < 0.05). According to previous studies [38], rCBV is associated with hypoxia-initiated angiogenesis that could lead to resistance to treatment. Moreover, our finding showed that higher tumor necrosis volume was associated with better survival, but higher rCBV at necrosis area prognosticated worse survival. Tumor necrosis rCBV indicates increased metabolic rate. It can be imaged by PET and MRS (Lactate peak) for further revaluation. Although non-significant, our results showed that higher rCBV values are correlated with worse survival in high perfusion ROI which could imply faster tumor growth.

The MD as a parameter of tissue cellularity [39] has a diagnostic performance. Our results showed that lower MD values correlated with worse survival, but significantly only in the short-term survival group (*p*-value < 0.05). Restricted diffusivity is due to higher tumor cellularity and a sign of higher proliferation. Several studies demonstrated that more aggressive gliomas have lower MD values [23,40], although a meta-analysis also showed that the MD value had an inverse correlation with cellularity in gliomas, this correlation was not consistent in all tumor types [19,20]. There was no significant correlation between FA and survival, but the FA values were significantly different in all ROIs.

Tumor contouring and segmentation is a challenging and important step because most work depends on manual delineation and validation slice by slice for each tumor. Although tumor segmentation was done by the k-means algorithm, it still needed to be verified by a neuroradiologist. This step is time-consuming, and inter- or intra-observer disagreement seems to be unavoidable. We proposed a method to determine different compartments in a heterogeneous GBM tumor using multi-modal MRI and investigated their effects on patient survival. The LMD-LrCBV ROI was described as the combination of the low MD and the low rCBV values in the CE region. Our results showed that lower MD and higher rCBV values correlated with poor survival in this ROI. Moreover, our results showed that the LMD-rCBV relative volume in short-term survival group was significantly larger than LMD-LrCBV relative volume in long-term survival group (*p*-value < 0.05) that may be a sign of higher cellularity and proliferation. GBM patients with short-term survival time tended to have a higher volume of the CE, low perfusion, and low diffusion in the CE region. Besides, a larger necrosis relative volume and HMD-LrCBV relative volume can indicate a better prognosis and survival time provided that rCBV is low. This can support the thesis that if most of the low perfusion compartment features high diffusion, the probability of the presence of other compartments and an aggressive tumor is smaller.

The great advantage of multimodal MRI analysis with a proper feature selection method is that the most effective features can be found and used to improve the performance accuracy of the machine learning model. In fact, our findings have clinical significance. The main purpose of the work was to develop an RF-RFE GBoost method that could identify influent features affecting the survival time and predict the survival time of GBM patients with high accuracy. The overall accuracy of the survival time prediction was approximately 75% for best features selected by RF-RFE and 55% without feature selection method, respectively. This work showed that advanced neuroimaging data in combination with unsupervised and supervised learning machines can provide an accurate result in predicting the survival time of GBM patients. There are patients who are expected to have poor outcomes due to GBM nature, but the prediction of a long survival time increases hope for improvement for the patients. Hence, more aggressive treatment is needed to improve survival time.

Other clinical significance of our study was that we included only GBM patients. GBMs are known with poor prognosis compared to other gliomas patients. In a similar study, Vergun et. al. [24] used multimodal images in combination with clinical and demographic variables to train SVM to predict patients’ outcome. Although their model was able to predict patients’ mortality with 80.7% accuracy; they included all grade types of the gliomas. Gliomas have significant different prognosis. Oligodandrogliomas as grade II are associated with good prognosis with median overall survival of 8 years. IDH-wild type diffuse astrocytomas are molecularly and clinically similar to GBM, and they have a poor prognosis (OS less than 2 years). IDH-mutant diffuse astrocytomas show intermediate survival time (OS bigger than 2 years) [41]. However, difference between their survivals is too large, and at least classification of all gliomas types into two classes might not to be a good idea.

There were some limitations of this study that needed to be considered. The main limitation was about the sample size. The main problem with a small dataset is interpretation of the results, and also the performance of the machine classifier depends on the sample size and characteristics of the sample. Conventional machine learning methods can well handle a small samples-size classification problem in subject level. Moreover, the feature selection method is one of the main techniques which can affect classifier performance and improve the classifier’s performance. Additionally, integration of structural and advanced MRI could help to better extract the most relevant parameters.

This study was a retrospective study, and data were collected in an individual center. It took several years to complete and collect data, and molecular analysis of CNS tumors was not readily available for all patients, and diagnosis was mostly based on histopathological analysis. Therefore, we were not able to consider molecular GBM information. However, methods to determine a GBM patient’s molecular subtypes or even tumor histopathology require an invasive biopsy or surgical resection. MRI as a rich source of patient’s information may serve as a noninvasive technique to determine molecular subtypes and survival time prediction of GBM patients. We aimed at predicting GBM patients’ survival time just based on their non-invasive pre-surgery MR images.

## 5. Conclusions

In conclusion, this work showed that the RF-RFE Gboost machine could identify useful features and predict the survival time of GBM patients with high accuracy. The clinical significance of this work is to provide a clinical tool in predicting complementary level of prognosis using only clinical MR images non-invasively. It may help surgeons and oncologists when deciding the treatment strategy.

## Figures and Tables

**Table 1:** Patients ‘characteristics and clinical information.

Demographics	
Age	
Age range	23–80 year
Average age	57.6 year
Gender	
Male	19
Females	10
Tumor located hemisphere	
Right	13
Left and bilateral	16
Survival time	
Short term survival < 15 month	16
Long term survival > 15 month	13

**Table 2:** Relationships between features and survival by univariate Cox regression and multivariate Cox regression.

**Relationships between Features and Survival by Univariate Cox Regression:**				
**Variable**	**Regression Coefficient (** $\beta_{i}$ **)**	**Hazard Ratio Exp (** $\beta_{i}$ **)**	**Confidence Level 95%**	***p*-Value**
Age	0.000	0.773	(0.947–1.056)	0.996
Sex(M)	−0.258	1.000	(0.259–2.302)	0.643
T1 in necrosis region	0.001	0.994	(1.000, 1.001)	0.04 *
T2 in necrosis region	0.000	1.002	(1.000, 1.001)	0.021 *
CBF in necrosis region	0.000	0.999	(0.998, 1.000)	0.013 *
rCBV in necrosis region	0.011	1.037	(1.014, 1.061)	0.001 *
rCBV in LMD-LrCBV in necrosis region	0.010	1.028	(1.007, 1.049)	0.007 *
rCBV in HMD-LrCBV in necrosis region	0.078	1.212	(1.041, 1.411)	0.013 *
rCBV in LMD-LrCBV in CE region	0.152	1.433	(1.064, 1.931)	0.018 *
**Relationships between Features and Survival by Multivariate Cox Regression**				
**Variable**	**Regression Coefficient** **(** $\beta_{i}$ **)**	**Hazard Ratio Exp (** $\beta_{i}$ **)**	**Confidence Level 95%**	***p*-Value**
T2 in necrotic region	0.000	1.000	(0.999, 1.001)	0.828
rCBV in LMD-LrCBV in CE region	0.001	1.001	(1.000, 1.001)	0.020 *
rCBV in necrosis region	0.113	1.120	(1.058, 1.186)	0.05 *

* The significance level is 0.05.

**Table 3:** Relative volume of ROIs in different groups.

	En (cm^3^)	Nec (cm^3^)	LMD-LrCBV (cm^3^)	HMD-LrCBV (cm^3^)	En-HrCBV (cm^3^)	Tumor Volume (cm^3^)
Short-termsurvival group	1.51 ± 0.45	2.07 ± 0.59	3.20 ± 0.73	1.01 ± 0.49	0.65 ± 0.37	6.02 ±5.1
Long-term survival group	1.26 ± 0.41	2.64 ± 0.65	2.47 ± 0.88	1.95 ± 0.77	0.42 ± 0.23	6.07 ± 5.4
*p*-value	0.190	0.049 *	0.032 *	0.003 *	0.178	0.536

* The significance level is 0.05.

**Table 4:** Performance GBoost classifier on the dataset with and without RF-RFE.

Classifier	Accuracy	Precision	Recall	F1-Measure	AUC	MCC
RF-RFE GBoost classifier	0.75	0.75	0.74	0.75	0.741	0.48
GBoost classifier (without RF-RFE)	0.58	0.58	0.58	0.58	0.58	0.16

## Data Availability

The dataset generated during and/or analyzed during the current study are not publicly available due to the clinical and confidential nature of the material but can be made available from the corresponding author on reasonable request.
